# Mpox outbreak in France: epidemiological characteristics and sexual behaviour of cases aged 15 years or older, 2022

**DOI:** 10.2807/1560-7917.ES.2023.28.50.2200923

**Published:** 2023-12-14

**Authors:** Catarina Krug, Arnaud Tarantola, Emilie Chazelle, Erica Fougère, Annie Velter, Anne Guinard, Yvan Souares, Anna Mercier, Céline François, Katia Hamdad, Laetitia Tan-Lhernould, Anita Balestier, Hana Lahbib, Nicolas Etien, Pascale Bernillon, Virginie De Lauzun, Julien Durand, Myriam Fayad, Investigation Team, Henriette De Valk, François Beck, Didier Che, Bruno Coignard, Florence Lot, Alexandra Mailles, Investigation Team (alphabetically), Lazare AGBAHOUNGBA, Amina AHAMADI, Ulviyya ALIZADA, Catherine AVENTINI, Patrick BAGUET, Elsa BALLEYDIER, Marie BARBA-VASSEUR, Anne-Sophie BARRET, Catherine BEC, Leila BEIKHEIRA, Anne BERNADOU, Julien BERRA, Nathalie BONNET, Elise BROTTET, Patrick CALEN, Carole DAGORNE, Sander DE SOUZA, Brigitte DEMMA, Audrey DIAVOLO, Florence DIDIER, Frédérique DORLEANS, Jacques EL KOURI, Céline ERIEAU, Julie FIGONI, Lucie FOURNIER, Sabrina FOURNIER, Diane FRANÇOIS, Olivier GLASS, Sophie GRELLET, Axel GRELLIER, Carine GRENIER, Jean-Paul GUYONNET, Valerie HENRY, Robin LE BARREAU, Clara LEYENDECKER, Louise LUAN, Yoann MALLET, Laurence MARAIS, Nathalie MATHEVET, Christine MEFFRE, Abdoul Djamal MOUKAILA, Ronan OLLIVIER, Damien OTT, Isabelle PARENT, Christine PERE, Laure PETIT-MADE, Valérie PONTIÈS, Philippe REBOUD, Laura REQUES, Patrick ROLLAND, Asma SAIDOUNI OULEBSIR, Lucie SAUTRON, Yassoungo SILUE, Ndeye Fatou SOW, Guillaume SPACCAFERRI, Ami YAMADA, Gabriel YUBERO, Aurélien ZHU-SOUBISE

**Affiliations:** 1 Santé publique France, The French Public Health Agency, Saint-Maurice, France; 2 ECDC Fellowship Program, Field Epidemiology path (EPIET), European Centre for Disease Prevention and Control (ECDC), Solna, Sweden; 3 These first authors contributed equally to this article; 4 Aix Marseille Univ, INSERM, IRD, Sciences Economiques et Sociales de la Santé & Traitement de l’Information Médicale (SESSTIM), Marseille, France; 5 Investigation Team members are listed below; 6 Centre de Recherche en Épidémiologie et Santé des Populations (CESP), Inserm U1018, Université Paris-Saclay, Université Paris-Sud, Université Versailles Saint-Quentin (UVSQ), Villejuif, France; 7 These authors contributed equally to this article

**Keywords:** France, Outbreak, Orthopoxvirus, Monkeypox, Mpox, Sexual and Gender Minorities, Sexual behaviour

## Abstract

**Background:**

Locally-acquired mpox cases were rarely reported outside Africa until May 2022, when locally-acquired-mpox cases occurred in various European countries.

**Aim:**

We describe the mpox epidemic in France, including demographic and behavioural changes among a subset of cases, during its course.

**Methods:**

Data were retrieved from the enhanced national surveillance system until 30 September 2022. Laboratory-confirmed cases tested positive for monkeypox virus or orthopoxviruses by PCR; non-laboratory-confirmed cases had clinical symptoms and an epidemiological link to a laboratory-confirmed case. A subset of ≥ 15-year-old male cases, notified until 1 August, was interviewed for epidemiological, clinical and sexual behaviour information. Association of symptom-onset month with quantitative outcomes was evaluated by t- or Wilcoxon tests, and with binary outcomes, by Pearson’s chi-squared or Fisher exact tests.

**Results:**

A total of 4,856 mpox cases were notified, mostly in Île-de-France region (62%; 3,025/4,855). Cases aged ≥ 15 years were predominantly male (97%; 4,668/4,812), with 37 years (range: 15–81) as mean age. Between May and July, among the subset interviewed, mpox cases increased in regions other than Île-de-France, and mean age rose from 35 (range: 21–64) to 38 years (range: 16–75; p = 0.007). Proportions of cases attending men-who-have-sex-with-men (MSM) meeting venues declined from 60% (55/91) to 46% (164/359; p = 0.012); median number of sexual partners decreased from four (interquartile range (IQR): 1–10) to two (IQR: 1–4; p < 0.001).

**Conclusion:**

Changes in cases’ characteristics during the epidemic, could reflect virus spread from people who were more to less behaviourally vulnerable to mpox between May and July, or MSM reducing numbers of sexual partners as recommended.

**Key public health message**

**What did you want to address in this study?**

Considering behavioural factors is key when implementing control measures during outbreaks of human-to-human transmitted infections, like those caused by monkeypox virus. In 2022, a mpox epidemic due to monkeypox virus occurred in Europe and France, mainly affecting men who have sex with men. We described the epidemiological characteristics of mpox cases in France, and changes, including in sexual behaviour, in a subset of male cases ≥ 15 years old.

**What have we learnt from this study?**

Between May and July 2022, the mean age of mpox cases increased in the subset of male cases ≥ 15 years old. In the same period, their median number of sexual partners decreased. This could mean that monkeypox virus spread from people who were more to less behaviourally vulnerable to mpox, as the epidemic progressed. Alternatively, this could reflect men who have sex with men reducing their numbers of sexual partners as recommended.

**What are the implications of your findings for public health?**

Our findings enabled practitioners and policymakers to monitor the mpox outbreak. They also supported efforts by the French government and non-governmental organisations to control the epidemic, by engaging populations behaviourally vulnerable to mpox. Whether behavioural changes noted in cases in our study generally apply to the French population of men who have sex with men needs further investigations.

## Introduction

Mpox is a zoonotic disease caused by monkeypox virus, a species belonging to the Orthopoxvirus genus in the Poxviridae family. The virus shares genomic and morphological features with variola virus, which is responsible for smallpox. There is little information in the literature about the wildlife reservoir of monkeypox virus, but rodents such as African rope squirrels (*Funisciurus* sp.), terrestrial giant pouched Gambian rats (*Cricetomys* sp.) and dormice (*Graphiurus* spp.) appear to be central in its epidemiology [1]. Transmission to humans can happen through contact with bodily fluids and mucous membranes of infected animals. Human-to-human transmission may also occur through close contact with an infected person (e.g. respiratory droplets, skin-on-skin, or sexual contact) and occasionally through fomites such as textile [2]. The incubation period ranges between 4 and 17 days [3].

Mpox is endemic in West and Central Africa [4], and before the year 2022, locally-acquired cases were rarely reported outside Africa [5]. Following the detection of clusters of non-travel-related mpox cases in Portugal and the United Kingdom (UK) in early May 2022, locally-acquired mpox cases were reported across Europe and North America [6,7]. As at 23 September 2022, there were more than 65,000 mpox cases reported worldwide [7], including more than 4,000 in France [8]. Over 95% of cases were men who have sex with men (MSM) [9-11]. Anogenital symptoms were frequent, suggestive of transmissibility during sexual contact [2,3].

Upon detection of the first locally-acquired cases in the UK, on 17 May 2022 the French surveillance system moved from routine mandatory notification of orthopoxvirus cases to an enhanced surveillance of mpox cases [8]. The objectives of this surveillance were to detect and isolate cases as well as to identify, trace and monitor their contacts. Cases were thus investigated and invited to name their at-risk contacts for contact-tracing, and at-risk contacts were offered post-exposure vaccination with smallpox vaccine from 25 May 2022 onwards [12].

The primary objective of the current study was to describe region of notification, age and sex of mpox cases detected through the enhanced mpox surveillance system until 30 September 2022 in France. In addition, this investigation aimed to provide epidemiological, clinical, and behaviour information on a subset of male cases aged 15 years and older, who occurred until 1 August 2022, to capture changes in their sexual behaviour as the epidemic progressed. A secondary objective was to describe associations between epidemiological characteristics and reported sex in the 3 weeks before symptom onset.

## Methods

### Mpox surveillance system

In France, mpox is a notifiable disease, like other orthopoxvirus diseases. Physicians and laboratories report cases to regional health agencies. During the mpox epidemic in 2022, regional health agencies and Santé publique France interviewed the reported cases using a standardised questionnaire exploring symptoms, contact with other cases, as well as travel history, sexual practices, and visits to MSM meeting venues. After 8 July 2022, this detailed questionnaire investigation was only administered to part of the cases, including one in five male cases (selected randomly) and all female and paediatric cases (below 15 years of age). For the other cases, a more limited amount of information (e.g. no travel history question, and fewer on sexual practices) was gathered. This change intended to reduce the investigation workload, as case numbers increased nationally. From 2 August onwards, questionnaires were completed only for female and paediatric cases [8].

### Participants and case definitions

This was an observational study of mpox cases reported through the French surveillance system.

For the overall descriptive epidemiology of the outbreak, all mpox cases notified until 30 September 2022 were included. Confirmed cases had a laboratory-confirmed monkeypox virus infection according to the national case definition, i.e. testing positive by real-time PCR for monkeypox virus or a generic orthopoxvirus. Non-laboratory-confirmed cases included anyone presenting a rash suggestive of mpox on any part of their body (including genital/perianal, oral) who also: (i) had an epidemiological link to a confirmed mpox case in the 3 weeks before symptom onset (i.e. probable case); or (ii) were MSM, or any person (regardless of gender or sexual orientation) who, in the 3 weeks before symptom onset, had two or more sexual partners, or had travelled to an endemic African country (i.e. possible case). The rash could be isolated (with no other symptoms), preceded by or accompanied with fever (> 38 °C), swollen lymph nodes (lymphadenopathy), or pain when swallowing (odynophagia).

To obtain epidemiological characteristics and data on sexual behaviours, a subset of male cases aged 15 years or older (age at which sexual maturity is considered established in France [13]) was investigated between 17 May and 1 August. A flowchart on how the cases were selected is presented in Supplementary Figure S1.

### Data collection

Physicians collected lesion swabs from suspected mpox cases. Initially, biological case confirmation was done by the national reference laboratory (in Brétigny sur Orge), but subsequently also by hospital laboratories, and private laboratories nationwide [14].

The subset of male cases interviewed until 1 August 2022 provided demographic, clinical and behavioural information that are detailed in Supplementary Table S1. In summary, information collected comprised region of notification, age and sex, human immunodeficiency virus (HIV) status, HIV pre-exposure prophylaxis (PrEP) use, clinical characteristics, sexual orientation and behaviour, and specific exposures in the 3 weeks before symptom onset including travel history, number of sexual partners, MSM venue attendance (clubs, bars, private parties, backrooms or saunas), practices of insertive or receptive anal sex, sadomasochism, and sexualised drug use (i.e. used before or during sex to sustain or enhance the experience), namely chemsex (also called ‘party and play’ (PnP) or ‘wired play’) and slam (intravenous drug injection). Questions on sexual behaviour were adapted from a previously validated survey [15]. MSM venue attendance was described in this study to understand the dynamics of the monkeypox virus spread over time; and questions on sadomasochism and on sexualised drug use were described due to the known relationship between practices leading to mucosal trauma and greater risk infection transmission during sex [16].

### Statistical analysis

We described quantitative variables with mean and range, or median and interquartile range (IQR), as appropriate, and binary variables with proportions. We also described variables by month of symptom onset and visually inspected graphs for any apparent trends. To evaluate associations between quantitative variables and month of symptom onset or reported sex, we ran a t-test or Wilcoxon test, as appropriate. To assess associations between binary variables and month of symptom onset or reported sex, we used Pearson’s chi-squared or Fisher exact tests, as appropriate. All analyses were done using Stata 17 (StataCorp, College Station, Texas, United States (US)).

## Results

### Descriptive epidemiology

**Figure 1:**
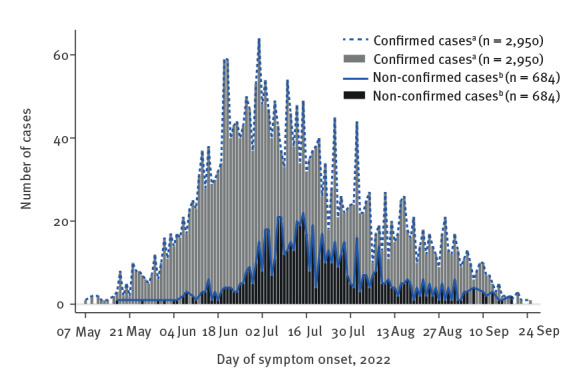
Confirmed^a^ (in grey bars and blue dashed line, n = 2,950) and non-laboratory-confirmed^b^ (in black bars and blue full line, n = 684) mpox cases by day of symptom onset, France, 7 May–25 September 2022^c^

A total of 4,856 mpox cases were notified in France between 17 May and 30 September 2022. Of these, 4,028 were laboratory-confirmed cases and 828 were non-laboratory-confirmed cases. The symptom onset dates of the cases occurred between 7 May and 25 September (Figure 1), and the number of cases peaked on 1 July with 79 cases. Cases with notification region information were mainly notified in the Île-de-France region (62%; 3,025/4,855), which includes Paris and its surroundings as depicted in Supplementary Figure S2. There were 20 children under 15 years old, including 12 boys and eight girls. Their mean age was 7 years (range: 2–14). Most cases aged 15 years or older were men (97%; 4,668/4,812), 125 (3%) were women, and 19 (<1%) did not specify their sex. Mean age was 37 years (range: 16–81; standard deviation (SD): 10) for men, 32 years (range: 15–66; SD: 11) for women, and 36 years (range: 24–56; SD: 10) for those with sex not specified.

Day of symptom onset was missing for 1,078 confirmed and 144 non-confirmed mpox cases.

^a^ Confirmed cases had a laboratory-confirmed monkeypox virus infection according to the national case definition, i.e. testing positive by real-time PCR for monkeypox virus or a generic orthopoxvirus.

^b^ Non-laboratory-confirmed cases included anyone presenting a rash evocative suggestive of mpox on any part of their body (including genital/perianal, oral) who also: (i) had an epidemiological link to a confirmed mpox case in the 3 weeks before symptom onset; or (ii) were men who have sex with men, or any person (regardless of gender or sexual orientation) who, in the 3 weeks before symptom onset, had two or more sexual partners, or had travelled to an endemic African country.

^c^ Symptom onset of cases ranged between 7 May and 25 September and notification dates ranged between 17 May and 30 September.

In Table 1, we describe characteristics of the subset of 2,216 male mpox cases aged 15 years or older with symptom onset up to 1 August 2022. About one third (35%; 401/1,148) of cases with available information were secondary cases who were either reported as a contact (n = 173), or who were not reported as such but turned out (after further investigation) to have been exposed to an individual with a monkeypox virus infection (n = 228). Of the secondary cases who were reported as a contact of a mpox case, 71% (123/173) indicated that the most probable transmission pathway was sexual.

**Table 1:** Epidemiological characteristics and clinical history of male mpox cases aged 15 years or older, France, 17 May–1 August 2022 (n = 2,216 cases)

Epidemiological characteristics	Level	Laboratory-confirmed cases			Non-laboratory-confirmed cases			All cases		
		Number	Denominator	%	Number	Denominator	%	Number	Denominator	%
**Type of case**	Index case	700	1,029	68.0	47	119	39.5	747	1,148	65.1
	Secondary case^a^	329	1,029	32.0	72	119	60.5	401	1,148	34.9
	Do not know	280	1,935	14.5	18	281	6.4	298	2,216	13.5
	Missing	626	1,935	32.4	144	281	51.3	770	2,216	34.8
**Probable transmission pathway** ^b^	Sexual	107	147	72.8	16	26	61.5	123	173	71.1
	Same household (non-sexual)	21	147	14.3	8	26	30.8	29	173	16.8
	Friend (non-sexual)	13	147	8.8	2	26	7.7	15	173	8.7
	Other (non-sexual)	6	147	4.1	0	26	0.0	6	173	3.5
**Travel to another country in the 3** **weeks before symptom onset**^c^	Yes	270	1,054	25.6	20	71	28.2	290	1,125	25.8
	No	784	1,054	74.4	51	71	71.8	835	1,125	74.2
	Do not know	76	1,500	5.1	8	169	4.7	84	1,669	5.0
	Missing	370	1,500	24.7	90	169	53.3	460	1,669	27.6
**MSM venue attendance in the 3** **weeks before symptom onset**^c,d^	Yes	503	1,034	48.7	23	65	35.4	526	1,099	47.9
	No	531	1,034	51.4	42	65	64.6	573	1,099	52.1
	Prefer not to say	11	1,500	0.7	0	169	0.0	11	1,669	0.7
	Missing	455	1,500	30.3	104	169	61.5	559	1,669	33.5
**Symptoms: rash** ^e^	Yes^f^	1,524	1,596	95.5	132	142	93.0	1,656	1,738	95.3
	No	72	1,596	4.5	10	142	7.0	82	1,738	4.7
**Symptoms: location of rash** ^g,h^	Genitals	792	1,480	53.5	73	127	57.5	865	1,607	53.8
	Peri-anal	582	1,480	39.3	54	127	42.5	636	1,607	39.6
	Face	560	1,480	37.8	43	127	33.9	603	1,607	37.5
	Palm	287	1,480	19.4	26	127	20.5	313	1,607	19.5
	Sole	185	1,480	12.5	10	127	7.9	195	1,607	12.1
	Other	873	1,480	59.0	76	127	59.8	949	1,607	59.1
	Missing^h^	44	1,524	2.9	5	132	3.8	49	1,656	3.0
**Symptoms: extracutaneous** ^g^	Fever (> 38 °C)	1,056	1,488	71.0	87	130	66.9	1,143	1,618	70.6
	Lymphadenopathy	1,033	1,508	68.5	84	128	65.6	1,117	1,636	68.3
	Myalgia	710	1,432	49.6	70	130	53.9	780	1,562	49.9
	Headache	646	1,385	46.6	63	130	48.5	709	1,515	46.8
	Throat ache	496	1,427	34.8	51	129	39.5	547	1,556	35.2
**Hospitalisation**	Yes	57	1,806	3.2	2	235	0.9	59	2,041	2.9
	No	1,749	1,806	96.8	233	235	99.2	1,982	2,041	97.1
	Missing	129	1,935	6.7	46	281	16.4	175	2,216	7.9
**Previous smallpox vaccination**	Yes	282	1,519	18.6	37	218	17.0	319	1,737	18.4
	No	1,237	1,519	81.4	181	218	83.0	1,418	1,737	81.6
	Missing	416	1,935	21.5	63	281	22.4	479	2,216	21.6
**Patient reported living with HIV**	Yes	390	1,550	25.2	29	139	20.9	419	1,689	24.8
	No	1,160	1,550	74.8	110	139	79.1	1,270	1,689	75.2
	Do not know	76	1,935	3.9	10	281	3.6	86	2,216	3.9
	Missing	309	1,935	16.0	132	281	47.0	441	2,216	19.9
**Immunosupression**	Yes	77	1,471	5.2	4	132	3.0	81	1,603	5.1
	No	1,394	1,471	94.8	128	132	97.0	1,522	1,603	95.0
	Do not know	92	1,935	4.8	13	281	4.6	105	2,216	4.7
	Missing	372	1,935	19.2	136	281	48.4	508	2,216	22.9
**HIV pre-exposure prophylaxis use** ^i^	Yes	723	1,116	64.8	58	104	55.8	781	1,220	64.0
	No	393	1,116	35.2	46	104	44.2	439	1,220	36.0
	Do not know	13	1,160	1.1	2	110	1.8	15	1,270	1.2
	Missing	31	1,160	2.7	4	110	3.6	35	1,270	2.8

HIV: human immunodeficiency virus; MSM: men who have sex with men.

^a^ Secondary cases were either immediately identified as a contact or turned out (after further investigation) to have been exposed to an infected individual without reporting this.

^b^ Among those who were identified as a contact; i.e. answered ‘Yes’ to the question ‘At the time of the onset of the symptoms, were you already being followed up as an at-risk contact of another probable or confirmed case?’. A probable case was defined, according to the national definition, as anyone presenting a rash evocative of mpox on any part of their body (including genital/perianal, oral) who also had an epidemiological link to a confirmed mpox case in the 3 weeks before symptom onset. A confirmed case had a laboratory-confirmed monkeypox virus infection according to the national case definition, i.e. testing positive by real-time PCR for monkeypox virus or a generic orthopoxvirus.

^c^ Among those who answered ‘No’ or ‘I do not know’ to the question ‘At the time of the onset of the symptoms, were you already being followed up as an at-risk contact of another probable or confirmed case?’. The definitions of probable and confirmed cases can be found in footnote b above.

^d^ Went to MSM bars, saunas, backrooms, or participated in punctual events (festival, gay pride) or lives with a person who did in the 3 weeks before symptom onset.

^e^ Cases whose symptoms and clinical signs were not documented (i.e. missing) were excluded from this analysis.

^f^ If the individual reported rash date, rash location or rash as one of the first symptoms.

^g^ A single case could have more than one symptom.

^h^ Missing if reported rash date or rash as one of the first symptoms, but no location recorded.

^i^ Among those who answered ‘No’ to ‘Living with HIV?’.

Among the 1,669 cases who were not being followed up as an at-risk contact of another case, 1,099 had data on visits to MSM meeting venues and 1,125 had data on travel. Among those with answers on MSM meeting venues, 48% (526/1,099) reported attending such places, while 26% (290/1,125) of cases with travel information reported travelling to another country in the 3 weeks before symptom onset. The most frequently visited countries included Spain (36%; 105/290), Germany (8%; 23/290), Belgium (7%; 21/290) and Italy (6%; 18/290).

Of cases whose clinical signs were documented, most reported a rash (95%; 1,656/1,738), fever (71%; 1,143/1,618) and lymphadenopathy (68%; 1,117/1,636). Only 3% (59/2,041) reported being hospitalised. None died. Among 1,737 persons with vaccination status available, 319 (18%) reported receipt of smallpox vaccination. One quarter (25%; 419/1,689) reported living with HIV, and 64% (781/1,220) of those not living with HIV reported using PrEP (Table 1).

### Sexual behaviour of men aged 15 years or older

The median number of sexual partners in the 3 weeks before symptom onset was three (IQR: 1–5; range: 0–100). Ninety-six per cent (1,745/1,817) of cases self-identified as MSM. The description of sexual behaviours during the 3 weeks before symptom onset, is presented in Table 2.

**Table 2:** Selected sexual behaviours in the 3 weeks before symptom onset of male mpox cases aged 15 years or older, France, 17 May–1 August 2022 (n = 2,216)

Sexual behaviour	Level	Laboratory-confirmed cases			Non-laboratory confirmed cases			All cases		
		Number	Total	%	Number	Total	%	Number	Total	%
**Number of sexual partners** ^a^	None	93	1,513	6.2	10	155	6.5	103	1,668	6.2
	One	338	1,513	22.3	48	155	31.0	386	1,668	23.1
	Two or more	1,082	1,513	71.5	97	155	62.6	1,179	1,668	70.7
	Do not know	62	1,935	3.2	8	281	2.9	70	2,216	3.2
	Missing	360	1,935	18.6	118	281	42.0	478	2,216	21.6
**Self-identified as MSM** ^a^	Yes	1,575	1,641	96.0	170	176	96.6	1,745	1,817	96.0
	No	66	1,641	4.0	6	176	3.4	72	1,817	4.0
	Did not wish to answer	30	1,935	1.6	2	281	0.7	32	2,216	1.4
	Missing	264	1,935	13.6	103	281	36.7	367	2,216	16.6
**Insertive anal sex** ^b^	Yes	757	971	78.0	33	53	62.3	790	1,024	77.2
	No	214	971	22.0	20	53	37.7	234	1,024	22.9
	Did not wish to answer	38	1,575	2.4	2	170	1.2	40	1,745	2.3
	Missing	566	1,575	35.9	115	170	67.7	681	1,745	39.0
**Receptive anal sex** ^b^	Yes	645	974	66.2	30	52	57.7	675	1,026	65.8
	No	329	974	33.8	22	52	42.3	351	1,026	34.2
	Did not wish to answer	40	1,575	2.5	2	170	1.2	42	1,745	2.4
	Missing	561	1,575	35.6	116	170	68.2	677	1,745	38.8
**Sadomasochism** ^b^	Yes	52	977	5.3	3	56	5.4	55	1,033	5.3
	No	925	977	94.7	53	56	94.6	978	1,033	94.7
	Did not wish to answer	24	1,575	1.5	1	170	0.6	25	1,745	1.4
	Missing	574	1,575	36.4	113	170	66.5	687	1,745	39.4
**Chemsex** ^b,c^	Yes	319	1,226	26.0	24	110	21.8	343	1,336	25.7
	No	907	1,226	74.0	86	110	78.2	993	1,336	74.3
	Did not wish to answer	22	1,575	1.4	1	170	0.6	23	1,745	1.3
	Missing	327	1,575	20.8	59	170	34.7	386	1,745	22.1
**Slam** ^b,d^	Yes	42	1,056	4.0	4	68	5.9	46	1,124	4.1
	No	1,014	1,056	96.0	64	68	94.1	1,078	1,124	95.9
	Did not wish to answer	18	1,575	1.1	1	170	0.6	19	1,745	1.1
	Missing	501	1,575	31.8	101	170	59.4	602	1,745	34.5

MSM: men who have sex with men.

^a^ Among all men.

^b^ Among MSM.

^c^ Sexualised drug use before or during sexual activity to sustain or enhance the experience; chemsex is also called Party and play (PnP) or wired play.

^d^ Sexualised drug injection before or during sexual activity to sustain or enhance the experience.

### Trends in demographic characteristics and sexual behaviour of male cases aged 15 years or older over time

**Figure 2:**
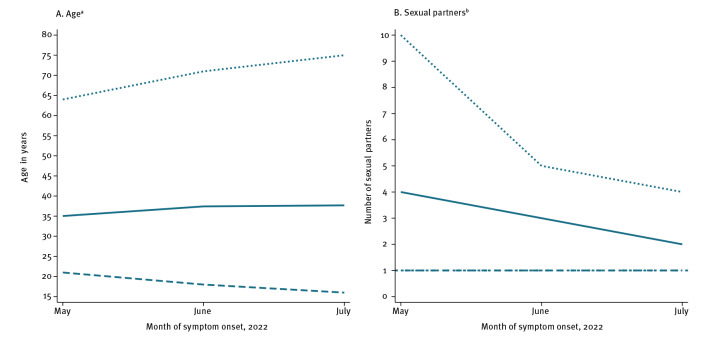
Evolution of (A) mean age, with range, of male mpox cases (n = 2,216) and (B) median number, with interquartile range, of sexual partners of male mpox cases (n = 1,449), by cases’ month of symptom onset, France, 7 May–31 July 2022

^a^ Inside the graph, the solid line represents the mean age of mpox cases, the dotted line the upper range, and the dashed line the lower range.

^b^ Inside the graph, the solid line represents the median number of sexual partners of mpox cases, the dotted line the 75^th^ percentile, and the dashed line the 25^th^ percentile.

**Figure 3:**
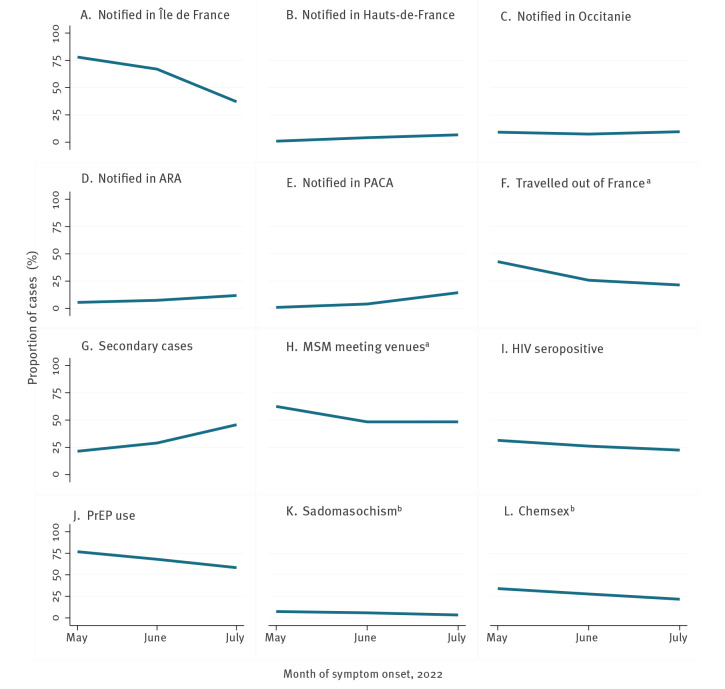
Evolution by month of symptom onset of (A−I) epidemiological characteristics and (J) PrEP use of male mpox cases, or (K−L) sexual behaviours among the subset of men who have sex with men, France, 7 May–31 July 2022 (n = 2,216)

ARA: Auvergne-Rhône-Alpes; HIV: human immunodeficiency virus; MSM: men who have sex with men; PACA: Provence-Alpes-Côte d’Azur; PrEP: HIV pre-exposure prophylaxis use among those who answered ‘No’ to ‘Patient living with HIV’.

^a^ Among those who answered ‘no’ or ‘I do not know’ to the question ‘At the time of the onset of the symptoms, were you already being followed up as an at-risk contact of another probable or confirmed case?’. A probable case, according to the national definition, was defined as anyone presenting a rash evocative of mpox on any part of their body (including genital/perianal, oral) who also had an epidemiological link to a confirmed mpox case in the 3 weeks before symptom onset. A confirmed case was a laboratory-confirmed monkeypox virus infection, i.e. testing positive by real-time PCR for monkeypox virus or a generic orthopoxvirus.

^b^ Among MSM in the 3 weeks before symptom onset. Chemsex was sexualised drug use before or during sexual activity to sustain or enhance the experience; chemsex is also called Party and play (PnP) or wired play.

The mean age of male cases increased slightly between May (35 years; range: 21–64) and July 2022 (38 years; range: 16–75; p = 0.007, Figure 2). In Figure 3, we observed a steep downward trend in the proportion of cases notified in Île-de-France, from 78% (85/109) in May to 37% (429/1,157) in July (p < 0.001). This coincided with an apparent increase in other regions such as Provence-Alpes-Côte d’Azur (from 1% (1/109) to 14% (167/1,160)), Auvergne-Rhône-Alpes (from 6% (6/109) to 12% (137/1,160)) and Nouvelle-Aquitaine (from 0% (0/109) to 5% (55/1,160)). The proportion of secondary cases increased from 18% (14/76) to 40% (100/252), whereas the proportion of cases reporting travelling to other countries decreased from 43% (39/91) to 21% (79/368; p < 0.001). In May, main destinations included Spain and Belgium (21/39). The proportion of cases visiting MSM meeting venues also decreased from 60% (55/91) in May to 46% (164/359) in July 2022 (p = 0.012). Finally, there were slight downward trends on the proportion of cases who were living with HIV (31% (33/105) in May to 22% (171/761) in July; p = 0.043) and using PrEP (77% (53/69) to 58% (330/566); p = 0.003).

Symptom onset of cases ranged between 7 May and 31 July, while notification dates ranged between 17 May and 1 August.

Only regions for which the number of cases notified exceeded 100 were depicted. Sample sizes for each graph were the following (A) 1,146; (B) 118; (C) 191; (D) 213; (E) 206; (F) 1,125; (G) 1,148; (H) 1,099; (I) 1,689; (J) 1,220; (K) 1,044; (L) 1,351.

Symptom onset of cases ranged between 7 May and 31 July, while notification dates ranged between 17 May and 1 August.

The median number of sexual partners decreased from four (IQR: 1–10) to two (IQR: 1–4; p < 0.001; Figure 2). The proportion of MSM remained constant over time (97% (102/105) to 95% (855/902); p = 0.293; not shown on the Figure). Figure 3 shows a non-significant decrease in trends of sadomasochistic practices (7% (6/81) to 3% (10/291); p = 0.116) and a decreasing trend on sexualised drug use (i.e. chemsex; 34% (32/94) to 22% (116/539); p = 0.009) among MSM. There was no apparent change in the frequency of sexualised drug injection (i.e. slam; data not shown on the figure) among cases (3% (2/58) to 3% (9/347); p = 0.911), but this may be due to the very small numbers.

### Associations between individuals’ characteristics and reported sex in the 3 weeks before symptom onset

The results on the associations between individuals’ characteristics and reported sex in the 3 weeks before symptom onset are presented in Supplementary Table S2. Individuals who reported no sexual partners also reported less international travelling (14% (9/65) vs 25% (240/965), p = 0.044) and less MSM venue attendance during the 3 weeks before symptom onset (20% (13/64) vs 49% (475/972), p < 0.001) than individuals who reported having had at least one sexual partner. There were also significant differences in the location of rash between both groups, which was more frequent on the face, palms of the hands or soles of the feet among individuals who reported no sexual partners. Moreover, individuals who reported no sexual partners also reported more often to be living with HIV (36% (33/91) vs 24% (340/1,423), p = 0.008) and less often to use PrEP (33% (17/52) vs 66% (690/1,048), p < 0.001) than individuals who reported having had at least one sexual partner. Whereas the proportion of peri-anal rash was greater among individuals who reported having had at least one sexual partner (40% (546/1,364)) than among those who reported no sexual partners (29% (25/86); p = 0.044), the proportion of rash in the genitals did not differ between the two groups (58% (50/86) vs 54% (734/1,364), p = 0.435). There were no differences in age (p = 0.128) and region of notification (p = 0.844), nor in the presence of rash and extracutaneous symptoms (Supplementary Table S2).

## Discussion

During the 2022 mpox outbreak in France, which started in May 2022, cases were initially concentrated in the Île-de-France region. Subsequently, in July 2022, their numbers increased in several other regions, particularly Provence-Alpes-Côte d’Azur and Auvergne-Rhône-Alpes. Like studies from the US [9] and Spain [10,17], we found that mpox in France disproportionately affected MSM. Results from interviewing a subset of male cases aged 15 years or older additionally indicate that between May and July 2022, some characteristics changed in this group, with a slight increase in mean age, as well as a decrease in proportions attending MSM meeting venues, living with HIV, and using PrEP. A reduction in the median number of sexual partners also occurred. Moreover, among MSM in the subset, there was a drop in the proportion of cases reporting sexualised drug use (i.e. chemsex).

These changes might be explained by an initial monkeypox virus transmission in Île-de-France, in May, among MSM with higher chances of exposure and infection (e.g. with more sexual partners), followed by spread of the virus nationwide, in June and July, among MSM with less chances of exposure and infection. The nationwide spread is supported by the increase in the proportion of secondary cases from May to July in the country, which potentially might have been driven by summer vacation or national tourism. Aside from noting that cases had more sexual partners in May, around half of male cases who reported travelling, had Spain and Belgium as their travel destinations. These travels could be associated to international gathering events/festivals such as the Maspalomas Pride in Spain (5 to 15 May 2022), or the fetish Darklands festival in Antwerp, Belgium (5 to 8 May 2022), as suggested by another study [18]. The older age found for cases in July might reinforce the idea that cases later in the epidemic were less behaviourally vulnerable to monkeypox virus infection, as older people may engage less in mass gathering events than younger ones [11]; nonetheless, it should be noted the increase in mean age was relatively small (35 to 38 years). On the other hand, the changes in population characteristics that were observed over the May to July period could also be due to behavioural changes in French MSM, who may have decreased their numbers of sexual partners, to reduce their chances of infection [19]; however, at the time of writing this manuscript, there was no literature supporting this hypothesis in France.

The proportion of individuals with genital rash did not differ between those who reported having had at least one sexual partner and those who reported no sexual partners in the 3 weeks before symptom onset. This could have resulted, when answering the question about number of sexual partners in the 3 weeks before symptom onset, from different interpretations of sexual intercourse (which was not defined during interviews): exclusively anal intercourse or including masturbation or oral sex. This hypothesis would also explain the greater proportion of individuals living with HIV among individuals who reported no sexual partners [20].

The proportion of people living with HIV was 25% among mpox cases in France, which is lower than reported in studies from the US (41%; 136/334) [9], Spain (40%; 73/181) [10], or in a systematic review across 16 countries (41%) [21]. This lower proportion may be explained by differences in the way the question was asked across different studies (in our study the question was ‘Patient living with HIV’, with response options ‘Yes’, ‘No’, ‘Do not know’, and we did not ask about serostatus), or differences in the selection of participants. For instance, in the US study [9], half of participants were recruited from one hospital providing services to many people living with HIV. The proportion of hospitalisations was also lower in our study (3%) than in the Spanish study (8%; 77/954) [10], and this may be explained by either a broader range of cases captured in our study and thus by a lower proportion of people living with HIV, which could potentially decrease the chances of hospitalisation. Alternatively results in the current study could be due to differences in in-hospital case isolation policies across countries – in France, cases were generally told to isolate at home.

Mpox cases in our study were more often living with HIV (25% vs 10%) and were more often taking PrEP (64% vs 19%) or using sexualised drugs (26% vs 9%) than French MSM participating in an online survey on sexual HIV preventive behaviours in 2019 [15]. This may be explained by a higher level of sexual activity among mpox cases than among French MSM participating in the survey, leading to increased exposure to the monkeypox virus and/or to more frequent access to healthcare services with a greater probability of being diagnosed.

The sex and age of cases in this study were similar to those previously reported in a systematic review including 4,222 mpox cases reported in 12 countries [22], or another one including 42,807 cases in 41 countries [23]. The proportions of cases reporting more than one sexual partner during the 3 weeks before symptom onset [9], or being MSM [22], or using sexualised drugs [10], were also similar to those of other studies. The most likely transmission pathway reported by 71% of secondary cases was sexual, which is also reflected by the zones where the rash was observed (53% genital and 40% perianal), and agrees with other reports [21,24,25]. Finally, the high proportion of PrEP use was also similar to that of other studies [19,26].

Our description of cases identified through the enhanced surveillance system enabled practitioners as well as policy makers to monitor and control the current mpox outbreak. Recommendations were made to reduce numbers of sexual partners [27]. Public health actions by the French government and non-governmental organisations encouraged involvement of populations behaviourally vulnerable to mpox in the control of the epidemic, such as MSM, people working in or attending sexual venues, and sex-workers. A hotline was established [28], and prevention messages engaging populations behaviourally vulnerable to mpox were disseminated through targeted social marketing campaigns. These messages were posted digitally on dating apps and community websites, passed on using community-based radio, or spread in MSM venues. They were also divulgated through communication material for healthcare centres and outreach efforts (posters, leaflets) [29]. In addition, contact-tracing, contact-warning, post exposure vaccination of at-risk contacts of cases [12,30] and preventive vaccination of populations behaviourally vulnerable to mpox using smallpox vaccines were implemented [8,31]. Public health measures also relied on interacting with healthcare professionals to increase their awareness and to enhance diagnostic testing [32].

Our study has several strengths, including the collection of valuable epidemiological information from a large number of cases at the national level through the national surveillance system. The data were regularly checked to ensure the validity of the questions and the completeness of the variables. The findings in this report are nevertheless subject to several limitations. First, our study may under-report mpox cases because of poor healthcare seeking behaviour (e.g. due to only mild symptoms, limited access to health services, lack of specific treatment, fear of stigmatisation). Under-reporting could have also occurred as physicians might have not recognised all cases (e.g. in case of atypical disease) or might have not reported all diagnosed cases. Also, in our overall sample, the 15- to 17-year-old age group comprised a total of 14 people, with 11 male and three female individuals. As a result, the subset of males aged 15 years or older included too few people aged under 17 years to conduct meaningful comparisons between adolescent and adults regarding epidemiological and behavioural characteristics. Second, as in any surveillance data, there is no conventional random sample, which may lead to under or overrepresentation of cases with particular characteristics (selection bias) and may also affect the underlying assumptions of statistical tests, leading to invalid estimates. Third, there was lack of information on the proportion among the cases of transgender people, who may be represented in the proportion of women or in the proportion of cases who neither identified as male or female. Fourth, there was a large amount of missing data particularly among non-laboratory confirmed cases. For instance, data were missing for 34% observations on MSM venue attendance and for 39% on anal intercourse or sadomasochistic practices in the 3 weeks before symptom onset. Reasons for missing data included the evolution of the surveillance system on 8 July, with instructions to question and investigate thoroughly only part of the cases. Other reasons were the interviewers’ high workload (many interviews), interviewers’ reluctance to ask sensitive questions, or respondents’ not wishing to answer (sensitive questions). Nevertheless, some interviewers reported that most cases were comfortable talking about sensitive topics. To minimise reluctance to answer sensitive questions, the questionnaire started with general questions, and gradually moved to more personal and intimate questions. Furthermore, all interviews were, to the extent possible, conducted privately, and the respondents could refuse to answer or stop the interview at any time.

### Conclusion

To conclude, between May and July 2022, we observed changes in mpox case characteristics associated to virus exposure such as a decrease in the number of sexual partners, and in MSM venue attendance. The observed changes could be due to a spread of monkeypox virus from populations more behaviourally vulnerable to mpox to those less behaviourally vulnerable; or to changes in behaviour of MSM, who followed the recommendations of prevention messages to decrease their numbers of sexual partners. These elements, together with the large-scale vaccination campaign in France, both as pre- and post-exposure prophylaxis [12,30], have most likely contributed to the decline of incidence observed later in summer 2022 [8]. Behavioural factors are essential to consider when implementing control measures during outbreaks of human-to-human transmitted infections such as those caused by monkeypox virus. To continue monitoring changes in the sexual behaviour of mpox cases, in October 2022 Santé publique France set up a questionnaire on sexual behaviours that physicians could propose to their patients at the time of mpox diagnosis and that could be self-completed (Meccdo, *Monkeypox Enquête comportementale complémentaire à la déclaration obligatoire*). Community-based participatory research studies are needed to evaluate risk perception and to adapt risk communication before, during and after outbreaks affecting specific populations, such as the 2022 mpox outbreak. Further such studies might help assess whether the behavioural changes observed among mpox cases in the current investigation also apply to the general French MSM population, and remain in the long term.

## Supplementary Data

397000 Supplementary Material
