# Supplementary material for: Mpox outbreak in France: epidemiological characteristics and sexual behaviour of cases aged 15 years or older, 2022
Source: Euro Surveill. 2023 Dec 14;28(50):2200923. doi: 10.2807/1560-7917.ES.2023.28.50.2200923 (PMC10831416; doi:10.2807/1560-7917.ES.2023.28.50.2200923)
Supplement: Supplementary Material [file 2200923_SupplementaryMaterial.pdf]

Supplementary materials for the article Mpox outbreak in France: epidemiological characteristics and sexual behaviour of cases aged 15 years or older, 2022

This supplementary material is hosted by *Eurosurveillance* as supporting information alongside the article entitled “Mpox outbreak in France: epidemiological characteristics and sexual behaviour of cases aged 15 years or older, 2022”, on behalf of the authors, who remain responsible for the accuracy and appropriateness of the content. The same standards for ethics, copyright, attributions and permissions as for the article apply. Supplements are not edited by *Eurosurveillance* and the journal is not responsible for the maintenance of any links or email addresses provided therein.

Figure S1 Flow diagram of cases

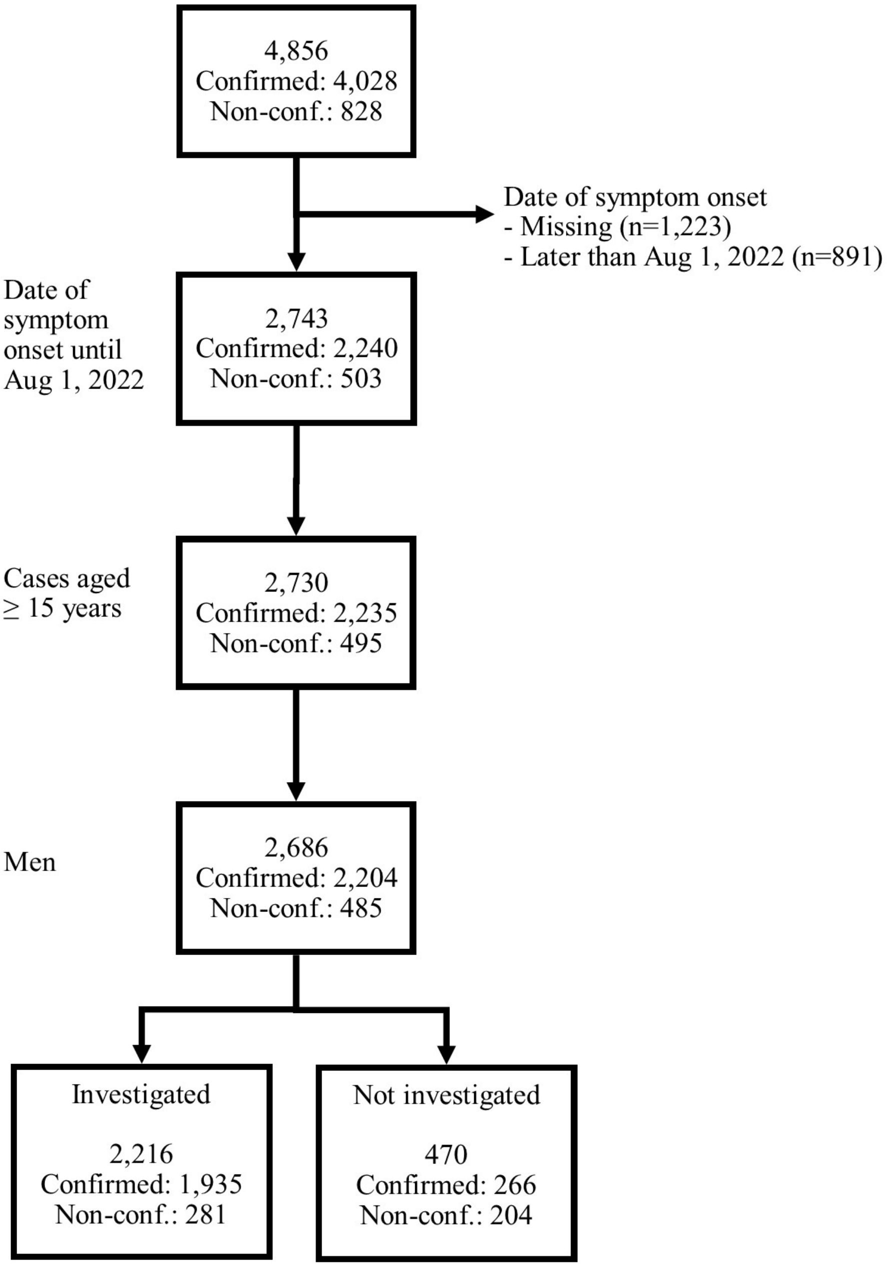

Note: Non-investigated cases were identified in our datasets as those with missing records for all the following variables: date of interview, HIV, immunosuppression, men who have sex with men, HIV pre-exposure prophylaxis use, pregnancy, symptoms variables, rash location, lymphadenopathy, fever, date of fever, headache, fatigue, myalgia, throat ache, cough, lesions, complications, contact with rodents after symptom onset, profession, known contact and multiple sex partners; 1,223 of cases with missing date of onset were excluded in the first step of the flow diagram, and 82% (1,015/1,223) of these were not investigated and would have been excluded at the latest stage of the flow diagram

Figure S2 Geographical distribution of case notification, France, 17 May–30 September 2022 (n = 4,855)

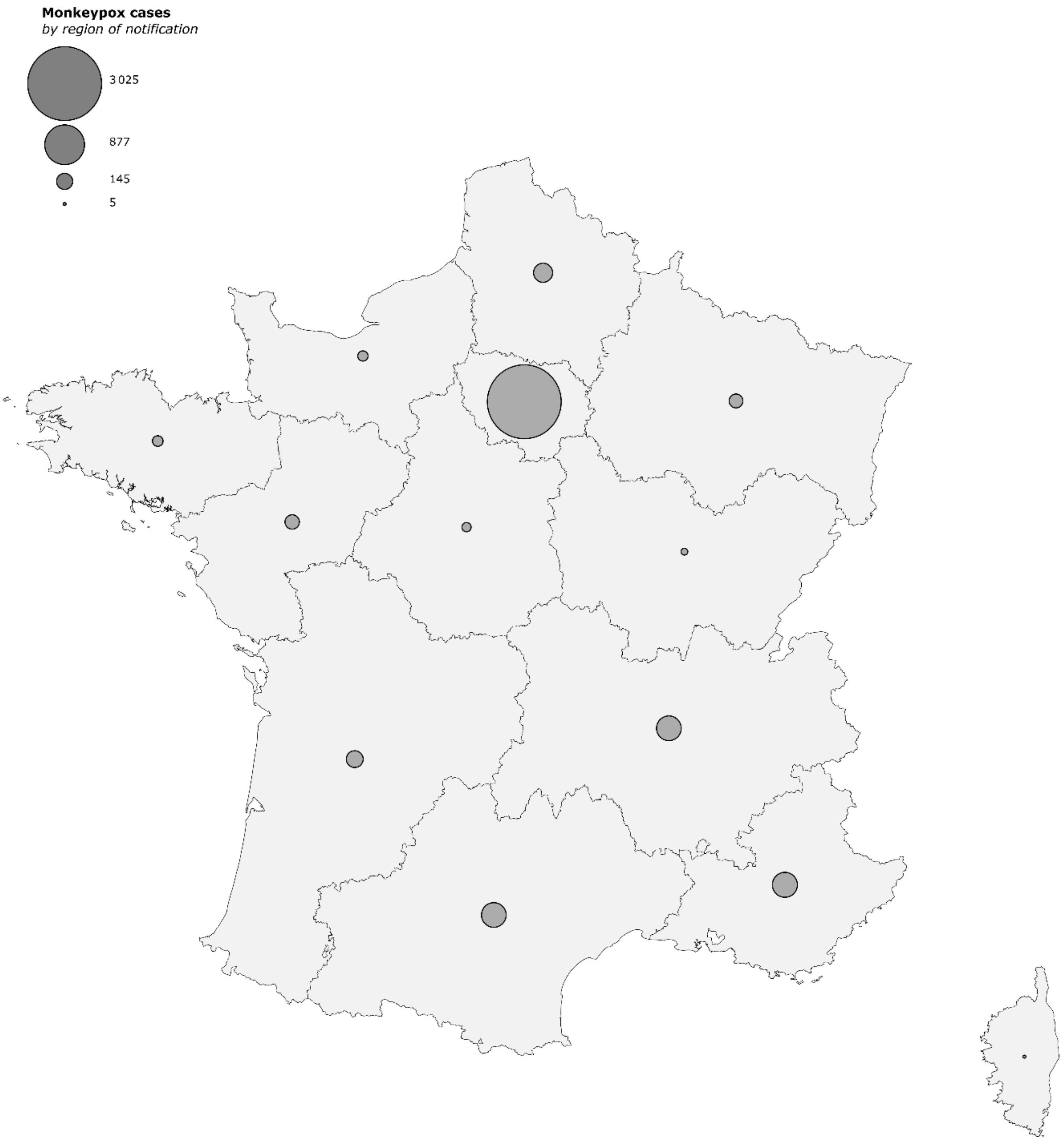

Note: The following cases are not depicted in the map: <5 in Guadeloupe, 9 in Martinique, <5 in La Réunion, <5 in Saint-Martin and 7 notified abroad

Table S1 Definition of outcomes

Outcome	Description	Population of interest (i.e. denominator)
Age	Age	All cases
Sex	Female, male, or other	All cases
Date of symptom onset	Date of symptom onset	All cases
Region of notification	Region of notification	All male cases
Travel in another country in the 3 weeks before symptom onset	Patient answered 'Yes' to the question 'Did you travel in another country in the 3 weeks before symptom onset?'	Answered 'Yes' or 'No' to the question and answered 'No' or 'I do not know' to the question 'At the time of the onset of the signs, were you already known and being followed up as an at-risk contact of another probable or confirmed case?'[a]
Known contact	Patient answered 'Yes' to the question 'At the time of the onset of the signs, were you already known and being followed up as an at-risk contact of another probable or confirmed case?'	Answered 'Yes' or 'No' to the question
Known contact non identified	Patient answered 'Yes' to the question 'Has frequented a person infected with the monkeypox virus but without being himself identified as an at-risk contact person (refusal of the index case to transmit his contacts, contact assessed as at negligible risk, confirmed index case abroad etc.)?'	Answered 'Yes' or 'No' to the question
Probable transmission pathway	The patient reported the most probable context of infection: Sexual, Same household (non-sexual), Friend (non-sexual), Other (non-sexual); other included	Answered 'Yes' to the question 'At the time of the onset of the signs, were you already known and being followed up as an at-risk contact of another probable or confirmed case?'
Type of case	Secondary case if answered 'Yes' to the question 'At the time of the onset of the signs, were you already known and being followed up as an at-risk contact of another probable or confirmed case?' and/or to the question 'Has frequented a person infected with the monkeypox virus but without being himself identified as an at-risk contact person (refusal of the index case to transmit his contacts, contact assessed as at negligible risk, confirmed index case abroad etc.)?';<br>Index case if answers 'No' to the first question and 'No'/'I do not know'/Missing to the second.	All male cases
Symptoms: rash	Rash if patient reported a rash date, location, or rash as first symptom	All male cases whose symptoms and clinical signs were documented (non missing)
Symptoms: location of rash	Patient reported a rash location: Genitals, Peri-anal, Face, Palm, Sole, Other	Patients who reported a rash location
Symptoms: extracutaneous	Patient reported other symptoms: Lymphadenopathy, Fever, Myalgia, Headache, Throat ache	All male cases
Hospitalisation	Hospitalisation due to monkeypox at any time before or at the time of notification	All male cases
Previous smallpox vaccination	Previous smallpox vaccination before 1980 or recently	All male cases
Living with HIV	Patient reported living with HIV	Answered 'Yes' or 'No'
Immunosuppression	Patient reported having immunosuppression	Answered 'Yes' or 'No' to the question
HIV pre-exposure prophylaxis use	Patient reported HIV pre-exposure prophylaxis use	Answered 'No' to 'Living with HIV'
MSM venue attendance	Patient answered 'Yes' to the question 'Did you go to MSM bars, saunas, backrooms, or did you participate in punctual events (festival, gay pride) or do you live with a person who did' in the 3 weeks before symptom onset	Answered 'Yes' or 'No' to the question and answered 'No' to the question 'At the time of the onset of the signs, were you already known and being followed up as an

		at-risk contact of another probable or confirmed case?’[a]
Number of sexual partners in the 3 weeks before symptom onset	Number of sexual partners in the 3 weeks before symptom onset	All male cases
Self-identifies as men who have sex with men (MSM)	Patient answered ‘Yes’ to the question ‘Do you define yourself as MSM (gay, bisexual, casual relationships with men)?’	Answered ‘Yes’ or ‘No’ to the question
Insertive anal sex	Patient answered ‘Yes’ to the question ‘Practiced insertive anal penetration in the 3 weeks before symptom onset’	Answered ‘Yes’ to the question ‘Do you define yourself as MSM (gay, bisexual, casual relationships with men)?’
Receptive anal sex	Patient answered ‘Yes’ to the question ‘Practiced receptive anal penetration in the 3 weeks before symptom onset’	Answered ‘Yes’ to the question ‘Do you define yourself as MSM (gay, bisexual, casual relationships with men)?’
Sadomasochism	Patient answered ‘Yes’ to the question ‘Did you participate in one or more SM/BDSM events (bondage, discipline, domination, submission, sado-masochism) in the 3 weeks before symptom onset?’	Answered ‘Yes’ to the question ‘Do you define yourself as MSM (gay, bisexual, casual relationships with men)?’
Chemsex	Patient answered ‘Yes’ to the question ‘Did you participate in one or more chemsex evenings (having consumed at least psychoactive product [cocaine, GHB/GBL, amphetamines, new synthetic products such as MDPV, 3-MMC, 4-MMC...] in a sexual context) in the 3 weeks before symptom onset?’	Answered ‘Yes’ to the question ‘Do you define yourself as MSM (gay, bisexual, casual relationships with men)?’
Slam	Patient answered ‘Yes’ to the question ‘Did you practice slam (i.e. used psychoactive products [or drugs] by injection during sexual intercourse) in the 3 weeks before symptom onset?’	Answered ‘Yes’ to the question ‘Do you define yourself as MSM (gay, bisexual, casual relationships with men)?’

[a] If someone did not know who was their contact person, travel history and men-who-have-sex-with-men venue attendance could perhaps identify certain exposure situations in the 3 weeks preceding the symptoms. These could allow for control measures to be implemented.

Note: According to the national definition, a probable case was defined as anyone presenting a rash evocative of mpox on any part of their body (including genital/perianal, oral) who also had an epidemiological link to a confirmed mpox case in the 3 weeks before symptom onset.

Table S2 Epidemiological characteristics and clinical history of male mpox cases aged 15 years or older who reported at least one or no sexual partners in the 3 weeks before symptom onset, France, 2022 (n=1,668)

Epidemiological characteristics and clinical history	Level	Reported at least one sexual partner in the 3 weeks before symptom onset (n=1,565)		Reported no sexual partners in the 3 weeks before symptom onset (n=103)		p[h]
		%	n/N	%	n/N	
Type of case	Index case	70.3	673/957	79.7	47/59	0.126
	Secondary case[a]	29.7	284/957	20.3	12/59	
	Do not know	14.8	231/1565	12.6	13/103	
	Missing	24.1	377/1565	30.1	31/103	
Probable transmission pathway[b]	Sexual	77.5	100/129	-	0/7	<0.001
	Same household (non-sexual)	10.9	14/129	71.4	5/7	0.001
	Friend (non-sexual)	7.0	9/129	28.6	2/7	0.100
	Other (non-sexual)	4.7	6/129	-	0/7	1.000
Travel to another country in the 3 weeks before symptom onset[c]	Yes	24.9	240/965	13.8	9/65	0.044
	No	75.1	725/965	86.2	56/65	
	Do not know	3.6	46/1,267	3.7	3/82	
	Missing	20.2	256/1,267	17.1	14/82	
MSM venue attendance[c,d]	Yes	48.9	475/972	20.3	13/64	<0.001
	No	51.1	497/972	79.7	51/64	
	Did not wish to answer	0.6	8/1,267	-	0/82	
	Missing	22.7	287/1,267	22.0	18/82	
Symptoms: rash[e]	Yes	96.7	1,390/1,438	93.6	87/93	0.136
	No	3.3	48/1,438	6.5	6/93	
Symptoms: location of rash[f]	Genitals	53.8	734/1,364	58.1	50/86	0.435
	Peri-anal	40.0	546/1,364	29.1	25/86	0.044
	Face	36.7	500/1,364	51.2	44/86	0.007
	Palm	19.1	260/1,364	27.9	24/86	0.045
	Sole	12.0	163/1,364	20.9	18/86	0.015
	Other	60.2	821/1,364	57.0	49/86	0.550
	Missing	1.9	26/1,390	1.2	1/87	-
Symptoms: extracutaneous	Fever	71.1	971/1,365	70.1	61/87	0.839
	Lymphadenopathy	68.6	948/1,383	62.5	55/88	0.238
	Myalgia	50.9	674/1,323	51.2	43/84	0.965
	Headache	47.6	611/1,285	48.2	39/81	0.917
	Throat ache	36.0	474/1,317	33.3	28/84	0.622
Hospitalisation	Yes	2.8	42/1,492	2.0	2/98	1.000
	No	97.2	1,450/1,492	98.0	96/98	
	Missing	4.7	73/1,565	4.9	5/103	

Previous smallpox vaccination	Yes	19.2	242/1,264	14.8	12/81	0.334
	No	80.9	1,022/1,264	85.2	69/81	
	Missing	19.2	301/1,565	21.4	22/103	
Patient reported living with HIV	Yes	23.9	340/1,423	36.3	33/91	0.008
	No	76.1	1,083/1,423	63.7	58/91	
	Do not know	2.4	37/1,565	3.9	4/103	
	Missing	6.7	105/1,565	7.8	8/103	
Immunosupression	Yes	4.9	66/1,344	8.0	7/88	0.208
	No	95.1	1,278/1,344	92.1	81/88	
	Do not know	3.3	51/1,565	3.9	4/103	
	Missing	10.9	170/1,565	10.7	11/103	
HIV pre-exposure prophylaxis use[g]	Yes	65.8	690/1,048	32.7	17/52	<0.001
	No	34.2	358/1,048	67.3	35/52	
	Do not know	1.0	11/1,083	1.7	1/58	
	Missing	2.2	24/1,083	8.6	5/58	

HIV, human immunodeficiency virus, MSM, men who have sex with men

[a] Secondary cases were either immediately identified as a contact or who turned out (after further investigation) to have been exposed to an infected individual without reporting this

[b] Among those who answered ‘Yes’ to the question ‘At the time of the onset of the signs, were you already known and being followed up as an at-risk contact of another probable or confirmed case?’

[c] Among those who answered ‘No’ or ‘I do not know’ to the question ‘At the time of the onset of the signs, were you already known and being followed up as an at-risk contact of another probable or confirmed case?’

[d] Went to MSM bars, saunas, backrooms, or participated in punctual events (festival, gay pride) or lives with a person who does in the 3 weeks before symptom onset

[e] Yes if reported rash date, location or rash as first symptoms; no if none of these and non-missing observation for the variable lymphadenopathy (i.e. to avoid including individuals with missing symptoms in the denominator)

[f] Missing if reported rash date or rash as first symptoms, but no location recorded

[g] Among those who answered ‘No’ to ‘Living with HIV’

[h] For the Pearson chi-squares or exact Fisher test
